# Liberality of a Foreign Critic

**Published:** 1852-10

**Authors:** 


*Liberality of a Foreign Critic.*—(From the British and Foreign and Medico-Chirurgical Review, for July, 1852.)—"Dr. Gross is quite correct in asserting that all the treatises on this subject as yet published in the English language, are mere outlines, which no one has attempted to render at all worthy companions to such works as those of Lawrence on 'Hernia,' Mackenzie on the 'Diseases of the Eye,' Budd on the 'Liver,' and Curling on the 'Testis.' It has remained for an American writer to wipe away this reproach; and so completely has the task been fulfilled, that we venture to predict for Dr. Gross's treatise (on the *urinary organs*) a permanent place in the literature of surgery, worthy to rank with the best works of the present age. Not merely is the matter good, but the getting-up of the volume is most creditable to Transatlantic enterprise; the paper and print would do credit to a first-rate London establishment; and the numerous wood-cuts which illustrate it, demonstrate that America is making rapid advances in this department of art.

"We have already expressed our high appreciation of Professor Gross's treatise; and it gives us much regret to feel compelled to state that the comparison of the two is, on almost every point, disadvantageous to Mr. Coulson. We acknowledge that we have tried his work by a high standard; but we think that we are fully justified in so doing, considering how long its author has been before the public as a surgical writer, and how high a position he evidently desires to attain. There is scarcely any point on which his work is not inferior to Dr. Gross's; and we have been continually led to feel, in our examination of it, how seriously defective it is, alike in method and in completeness; so that its perusal has left us with the conviction, that scarcely any one subject has been satisfactorily treated, scarcely any point of pathology or practice fairly and fully disposed of. We can assure Mr. Coulson that we state this with regret, and with no other motive than that which arises from our desire to discharge our critical duty faithfully and impartially. That our verdict will be confirmed by any competent judge, who may take the same pains that we have done in the comparison of the two works before us, we have not the smallest doubt; and we have even a sufficiently good opinion of Mr. Coulson's own candor, to believe, that if he will examine Professor Gross's treatise for himself, he will admit our case to be so strong, that he will not feel it necessary to impute to us any bias to his disadvantage."

